# Physiological characterization of a new thermotolerant yeast strain isolated during Brazilian ethanol production, and its application in high-temperature fermentation

**DOI:** 10.1186/s13068-020-01817-6

**Published:** 2020-10-27

**Authors:** Cleiton D. Prado, Gustavo P. L. Mandrujano, Jonas. P. Souza, Flávia B. Sgobbi, Hosana R. Novaes, João P. M. O. da Silva, Mateus H. R. Alves, Kevy P. Eliodório, Gabriel C. G. Cunha, Reinaldo Giudici, Diele P. Procópio, Thiago O. Basso, Iran Malavazi, Anderson F. Cunha

**Affiliations:** 1 Genetic and Evolution Department, Universidade Federal de São Carlos (UFSCar), São Carlos, SP 13565-905 Brazil; 2 Chemical Engineering Department, Escola Politécnica, Universidade de São Paulo (USP), São Paulo, SP 05508-010 Brazil

**Keywords:** *Saccharomyces cerevisiae*, Thermotolerant yeast, Stress resistance, Fermentation yields, Brazilian ethanol production

## Abstract

**Background:**

The use of thermotolerant yeast strains can improve the efficiency of ethanol fermentation, allowing fermentation to occur at temperatures higher than 40 °C. This characteristic could benefit traditional bio-ethanol production and allow simultaneous saccharification and fermentation (SSF) of starch or lignocellulosic biomass.

**Results:**

We identified and characterized the physiology of a new thermotolerant strain (LBGA-01) able to ferment at 40 °C, which is more resistant to stressors as sucrose, furfural and ethanol than CAT-1 industrial strain. Furthermore, this strain showed similar CAT-1 resistance to acetic acid and lactic acid, and it was also able to change the pattern of genes involved in sucrose assimilation (*SUC2* and *AGT1*). Genes related to the production of proteins involved in secondary products of fermentation were also differentially regulated at 40 °C, with reduced expression of genes involved in the formation of glycerol (*GPD2*), acetate (*ALD6* and *ALD4*), and acetyl-coenzyme A synthetase 2 (*ACS2*). Fermentation tests using chemostats showed that LBGA-01 had an excellent performance in ethanol production in high temperature.

**Conclusion:**

The thermotolerant LBGA-01 strain modulates the production of key genes, changing metabolic pathways during high-temperature fermentation, and increasing its resistance to high concentration of ethanol, sugar, lactic acid, acetic acid, and furfural. Results indicate that this strain can be used to improve first- and second-generation ethanol production in Brazil.

## Background

Currently, Brazil is the second ethanol producer worldwide using sugar cane as a fermentative substrate, since its high sucrose concentration is suitable for sugar and ethanol production [1–4]. During ethanol production, the wort, composed by molasses from sugar production and sugar cane juice, is mixed with yeast in high cell densities (8–22% v/v). The fermentation occurs in approximately 6–8 h with high ethanol yield at the end of fermentation (90–92%) [3, 4]. An important particularity within the Brazilian fermentation process yeast is the recycling process of yeast achieved by centrifugation followed by acid treatment in sulfuric acid (pH 2–2.25 for 2–3 h) used to reduce bacteria contamination during harvest. Fermentation occurs between 28 and 35 °C, and the maintenance of this temperature during the summer is only possible through water cooler use in fermentation tanks. Although this cooling process is crucial to maintain the temperature within an ideal range not exceeding 35 °C, it is costly and requires large quantities of water [5].

In addition to temperature, several other stressing conditions such as pH variation, osmotic stress, contamination by bacteria, and wild yeasts can affect the fermentative process [5]. Therefore, the production process should be performed using strains able to survive in all of these conditions in the fermentation tank. In Brazil, since the beginning of the 1990s, yeasts strains have been investigated to improve ethanol production. Several strains that naturally adapted to the sugar cane fermentation process used in Brazil were isolated and broadly used in several mills. The producers have mainly employed two industrial yeast strains known as CAT-1 and PE-2 whose genomes were sequenced in 2009 [2, 6–9].

Although these strains have had high performance for several years, the constitution regarding Brazilian fermentation substrates has been changing throughout the last years, especially after sugar cane burning was prohibited by law. As a result, there was a lower persistence of these strains in the fermentation tanks during the whole harvest that were then replaced by wild yeasts in several mills in Brazil [5]. Otherwise, CAT-1 and PE-2 were not able to ferment appropriately above 35 ºC, reducing their viability [10].

Aiming to maintain productivity or even improve the Brazilian fermentation process, our research group has been isolating new strains from distilleries since 2009 to identify new *Saccharomyces cerevisiae* strains that can adapt to the new conditions or that have new characteristics as ethanol, sugar and thermotolerance [5].

Here, we demonstrate the identification, and physiological and molecular characterization of a new thermotolerant *S. cerevisiae* strain that is able to grow at 40 °C while generating high ethanol yields under the aforementioned conditions. This strain has also shown important changes in the gene expression pattern in pathways involved in fermentation efficiency, which can provide information about its thermotolerant phenotype and associated fermentative performance. In addition, this strain is resistant to the stressors produced during the first- and second-generation ethanol production, highlighting the attributes of this strain for employment in high-temperature fermentation, endeavoring improvements in the Brazilian ethanol production chain.

## Results and discussion

### Isolation, identification and molecular genotyping of a thermotolerant yeast strain for use in high-temperature fermentation for ethanol production

**Fig. 1:**
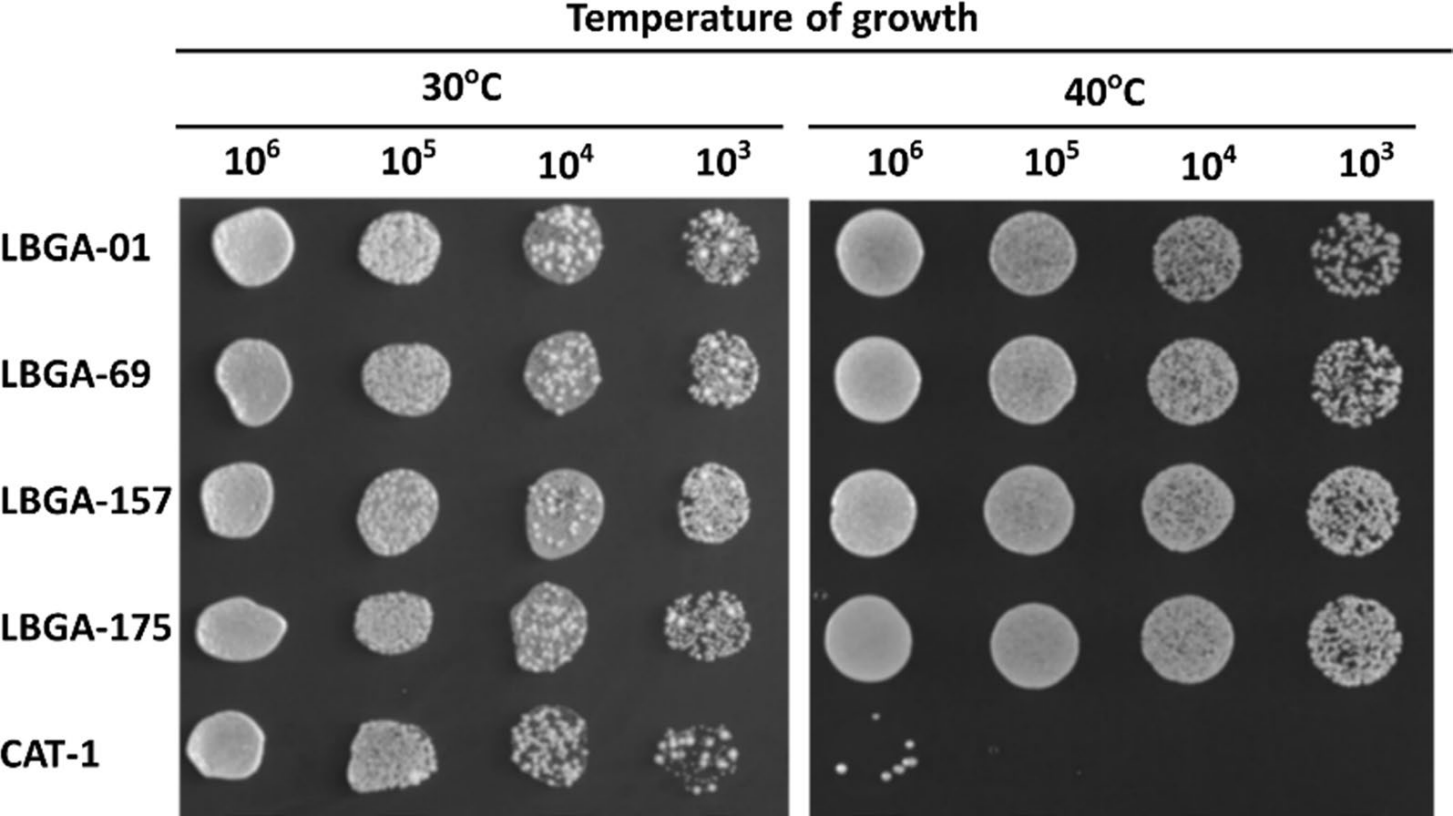
Isolation of thermotolerant yeasts from Brazilian ethanol production. Four thermotolerant yeasts have been identified as LBGA-01, LBGA-69, LBGA-157, and LBGA-175. Industrial strain CAT-1 was used as a non-thermotolerant strain control. Cells were grown overnight in YPD medium, and cell concentration was adjusted to 10^6^ cells/ml. Tenfold serial dilutions were spotted in YEPD solid media. Cells were incubated at 30 and 40 °C for 2 days. CAT-1 showed poor growth at 40 °C, and the four strains had the same pattern of growth at 30 and 40 °C

To obtain specialized yeast for ethanol production in Brazil, several attributes need to be considered and evaluated, such as ability to produce high yields of ethanol, high cell viability, and tolerance to stressors produced during ethanol production. Another important attribute is growth temperature. In Brazilian ethanol production, the strains currently used ferment at temperatures between 28 and 33 °C, so mills need water coolers to maintain temperature. The use of thermotolerant yeasts could circumvent this problem, improving ethanol production. The Laboratory of Biochemistry and Applied Genetics of the Federal University of São Carlos (LBGA-UFSCar) has been isolating yeasts from ethanol production since 2009, which have been deposited in the LBGA strain collection. We used this collection to screen for isolates that can grow at higher temperatures, above temperatures currently used in ethanol fermentation plants in Brazil. Within the approximately 300 strains, four isolates have been identified as thermotolerant since they are able to grow and display similar fitness at both 30 and 40 °C, unlike the industrial strain CAT-1 that is unable to growth at high temperatures [10]. These yeasts were identified as LBGA-01, LBGA-69, LBGA-157, and LBGA-175, respectively (Fig. 1).

To identify the strains, amplification of ITS [11] and a genotyping test were carried out. Results showed that the four strains were genetically distinct from one another, and also from the industrial strain CAT-1. The ITS analysis indicated that only LBGA-01 and LBGA-69 presented the amplification of a 900 bp, used to identify the strain as a possible *Saccharomyces* [11, 12]. LBGA-157 and LBGA-175 showed a different pattern of amplification, suggesting that these yeasts are non-*Saccharomyces* strains, but possibly wild contaminating strains (see Additional file 1). To confirm this prediction, ITS amplicons were sequenced. We confirmed through the BLAST analyses (see Additional file 2: Table S1) that LBGA-01 and LBGA-69 strains are *S. cerevisiae* isolates, while LBGA-157 and LBGA-175 strains are *Kluyveromyces marxianus* yeasts.

### Thermotolerant LBGA strains present superior fermentation performance at 40 °C in comparison to the industrial strain at 30 °C

**Fig.2:**
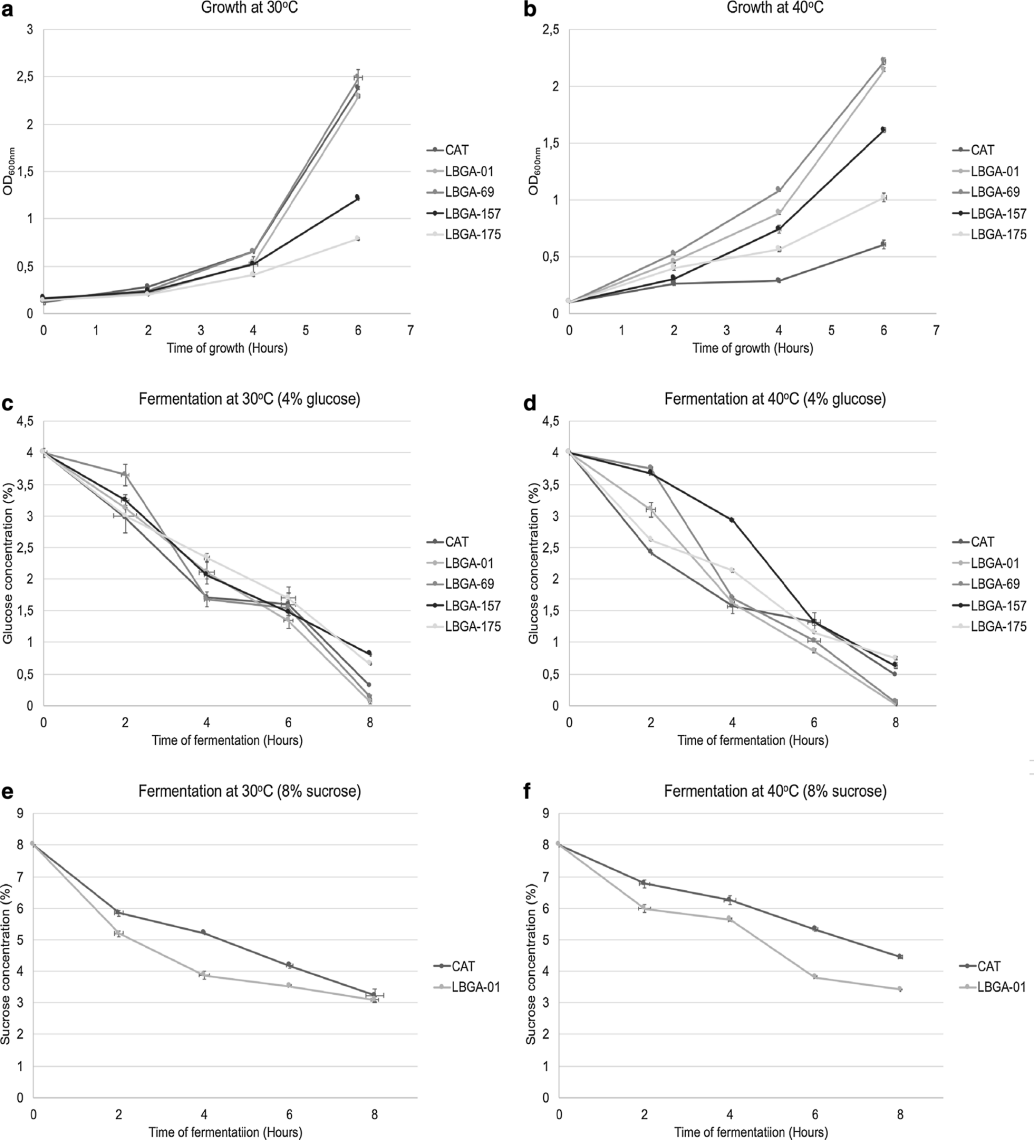
Comparison of growth and fermentation of isolated yeasts. The growth of yeasts (at 30 °C and 40 °C) was obtained by optical density at 600 nm (**a,** **b**). Fermentation rate at 30 °C and at 40 °C was evaluated with 4% of glucose (**c, d**) and 8% of sucrose (**e**, **f**) to be consumed through time

Although only the strains LBGA-01 and LBGA-69 were identified as *S. cerevisiae*, LBGA-157 and LBGA-175 were also included in cell growth and fermentation tests at 30 and 40 °C, since *Kluyveromyces* strains are also known to ferment at high temperatures [13]. Results from cell proliferation growth at 30 °C showed that the strains LBGA-01 and LBGA-69 have the same growth profile as the industrial strain CAT-1. However, when subjected to growth at 40 °C, former strains present higher growth rates than the industrial one. This result was already expected since CAT-1 has already not shown good performance when subjected to high temperatures [9, 10, 14]. The strains LBGA-157 and LBGA-175 showed slower growth rates at both temperatures. In fact, the strains LBGA-01 and LBGA-69 showed a similar growth profile in both temperatures, indicating they have a strong thermotolerant phenotype (Fig. 2a, b). As mentioned above, strains have to present excellent fermentation characteristics and a good conversion of sugar to ethanol in addition to thermotolerant growth to be suitable for industrial use. In ethanol production process, industrial yeasts convert substrate within the first hours of fermentation, thus converting all available sugar to ethanol after approximately 4–6 h. To evaluate the fermentative potential of isolated yeasts, we first performed a fermentation experiment using 4% of glucose and all the isolates. Results showed that LBGA-01 and LBGA-69 had a pattern similar to the industrial strain CAT-1 in fermentation conducted at 30 °C, and had superior performance at 40 °C. Strains LBGA-157 and 175 presented low performance in both temperatures (Fig. 2c, d). The yeasts used during ethanol fermentation are subjected to high concentrations of sugar, and are therefore constantly exposed to osmotic stress. Since LBGA-01 showed good fermentation performance and presented a slight advantage over LBGA-69 in glucose fermentation (Fig. 2c, d), we also conducted fermentative tests using 8% of sucrose concentration to simulate the conditions widely used in standard ethanol production in Brazil, in which sucrose is used as fermentable sugar. In comparison to the CAT-1 industrial strain, LBGA-01 showed a better performance in both temperatures with a clear superiority at 40 °C (Fig. 2e, f). In fermentation assays at 30 °C, both strains demonstrated a similar pattern. However, at 40 °C, LBGA-01 strain converted 57.5% of the initial sugar while the industrial strain converted 45% (Fig. 2f). Regarding the expected yields for a sugar cane plant, these results are considerable from an economic point of view, since the LBGA-01 strain converted 12.5% more sugar than the CAT-1 industrial strain under stress conditions. It is worth mentioning that the use of thermotolerant yeast operating under stress conditions with good fermentation rates can represent a significant increase in ethanol production in the plant. Other advantages are a decrease in contamination with wild yeasts and bacteria, as well as in water usage to control temperature in fermentation tanks, consequently reducing energy costs while contributing to environmental issues.

### LBGA-01 is resistant to stressors produced during 1G and 2G ethanol production process

**Fig. 3:**
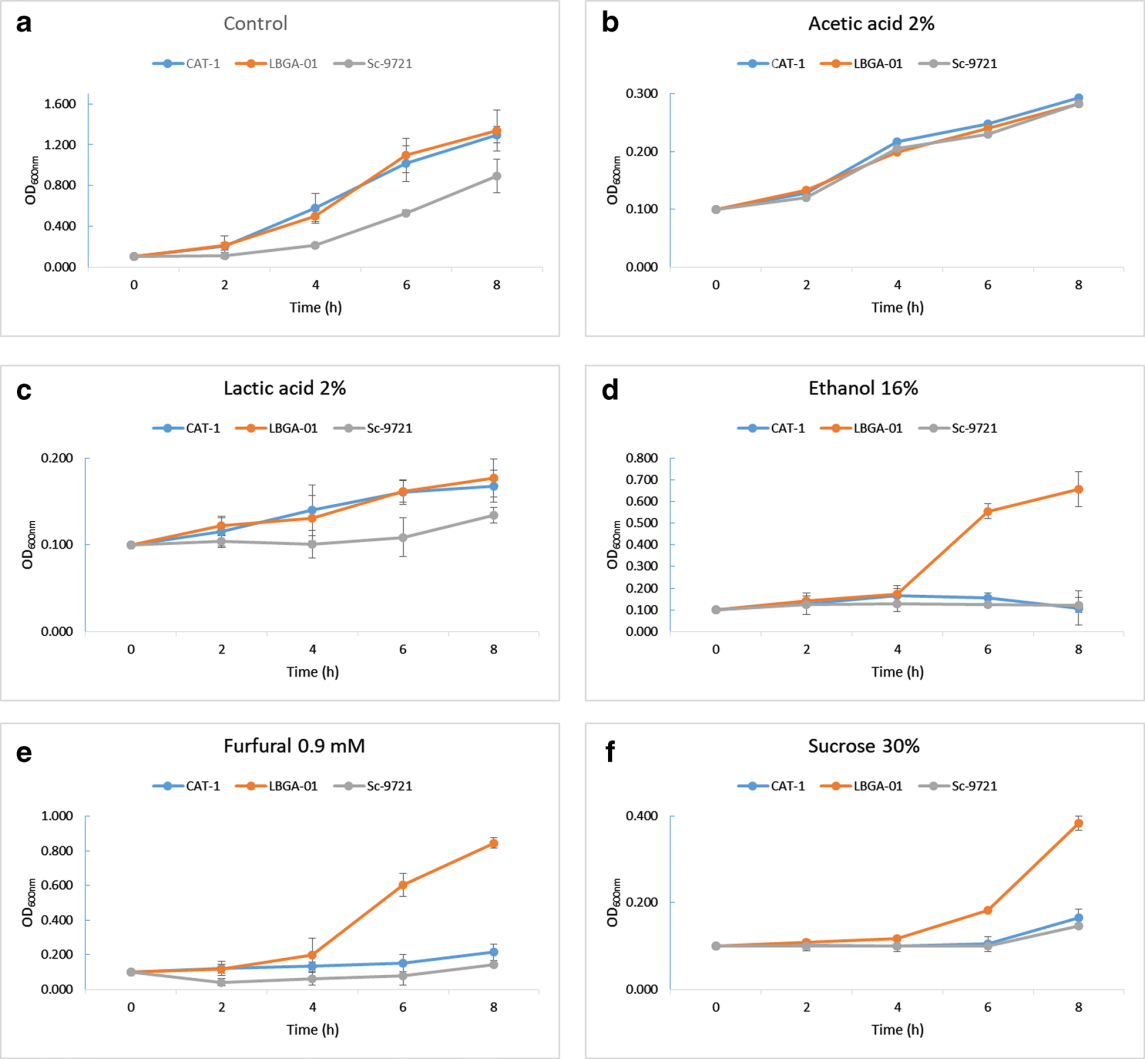
Viability analysis of thermotolerant yeast LBGA-01 compared to industrial (CAT-01) and haploid (Sc-9721) strains under different concentrations of 1G and 2G ethanol production stressors. The test lasted 8 h at 30 °C and 180 rpm, an aliquot of the culture medium was collected every 2 h and the absorbance was measured at OD_600_. The experiments were carried out in triplicate

Yeasts withstand constant stressing conditions during the Brazilian fermentation process to produce first (1G) and second (2G)-generation ethanol, directly resulting in a decrease of final yield. To evaluate tolerance of the LBGA-01 strain, we analyzed its growth and survival under different concentrations of stressors such as ethanol, sugar, lactic acid, acetic acid, HMF and furfural (the latter three being inhibitors of the 2G ethanol production process). Results were compared to the industrial strain CAT-1 and the laboratory haploid strain Sc-9721. The concentration of each stressor was established according to the literature, as described in the methodology section. Results obtained through growth curves in liquid cultures showed that the LBGA-01 strain is more resistant in most tested stressors (Fig. 3).

The overall positive results exceeded the initial expectations, especially regarding the presence of 2% acetic acid. We observed that the LBGA-01 strain showed similar resistance to CAT-1 strain and Sc-9721 laboratory strain. When subjected to 4% acetic acid, all strains suffered stress and had their growth inhibited (data not shown). In the lactic acid test (4%), LBGA-01 and CAT-1 strains were also more resistant than Sc-9721 laboratory strain. This indicates that this yeast would be resistant under conditions of contamination by *Bacillus* spp or other lactic acid-producing bacteria [15], and not affected during alcoholic fermentation. However, a wider range of concentration needs to be tested to determine in which range of lactic acid concentration the LBGA-01 strain can survive. We observed higher resistance of LBGA-01 in comparison to CAT-1 and laboratory Sc-9721 strains at 16% of ethanol. In this trial, the control laboratory and industrial strains were drastically affected by ethanol stress. When subjected to high concentrations of sucrose (30%), the LBGA-01 strain was also more resistant than CAT-1 and Sc-9721 strains. This suggests that LBGA-01 could ferment in a high osmolarity medium that will produce high ethanol yields, and consequently increase ethanol production. For the HMF test, no strain grew in the lowest evaluated concentration of 40 mM (data not shown). Growth tests with furfural showed that the LBGA-01 strain is more resistant than the industrial and laboratory strains at the tested concentrations of 0.9 mM. Furfural is a potent inhibitor of *Saccharomyces* during lignocellulosic fermentation [16]. For this reason, the thermotolerance phenotype together with the furfural resistance could be an important feature for the production and improvement of 2G ethanol, as described for LBGA-01. Together, these results demonstrate the robustness of the LBGA-01 strain in the presence of several stressors during the fermentation process, making it a potential strain for improving the 1G and 2G ethanol production.

### Transcriptional responses of LBGA-01 under high-temperature fermentation conditions

**Fig. 4:**
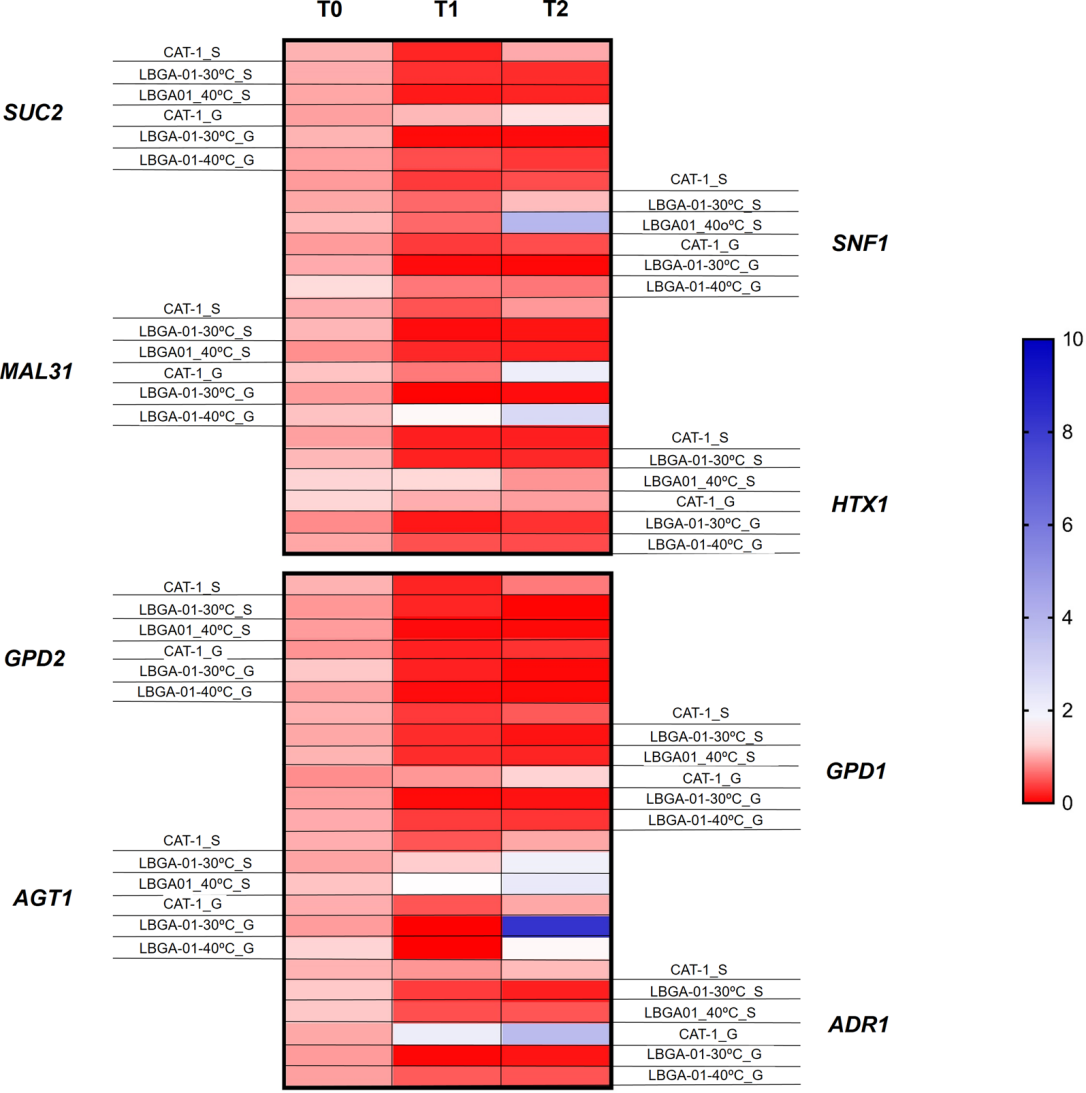
Expression profile of genes involved in fermentation efficiency of LBGA-01 fermented at high temperature. The fermentation of LBGA-01 and industrial CAT-1 strains was performed at 30 °C. Glucose (*G*) and sucrose (*S*) was used as carbon sources. The bar colors represent gene expression profile (red: low expression; blue: high expression)

To better understand the metabolic changes in LBGA-01 strain during high-temperature fermentation, the expression of genes involved in fermentation efficiency, membrane biosynthesis and sucrose assimilation were evaluated using qPCR. Results were compared with the industrial yeast CAT-1 at both fermentation temperatures (30 °C and 40 °C). The mRNA levels for genes involved in the efficiency of fermentation are summarized in Fig. 4.

**Fig. 5:**
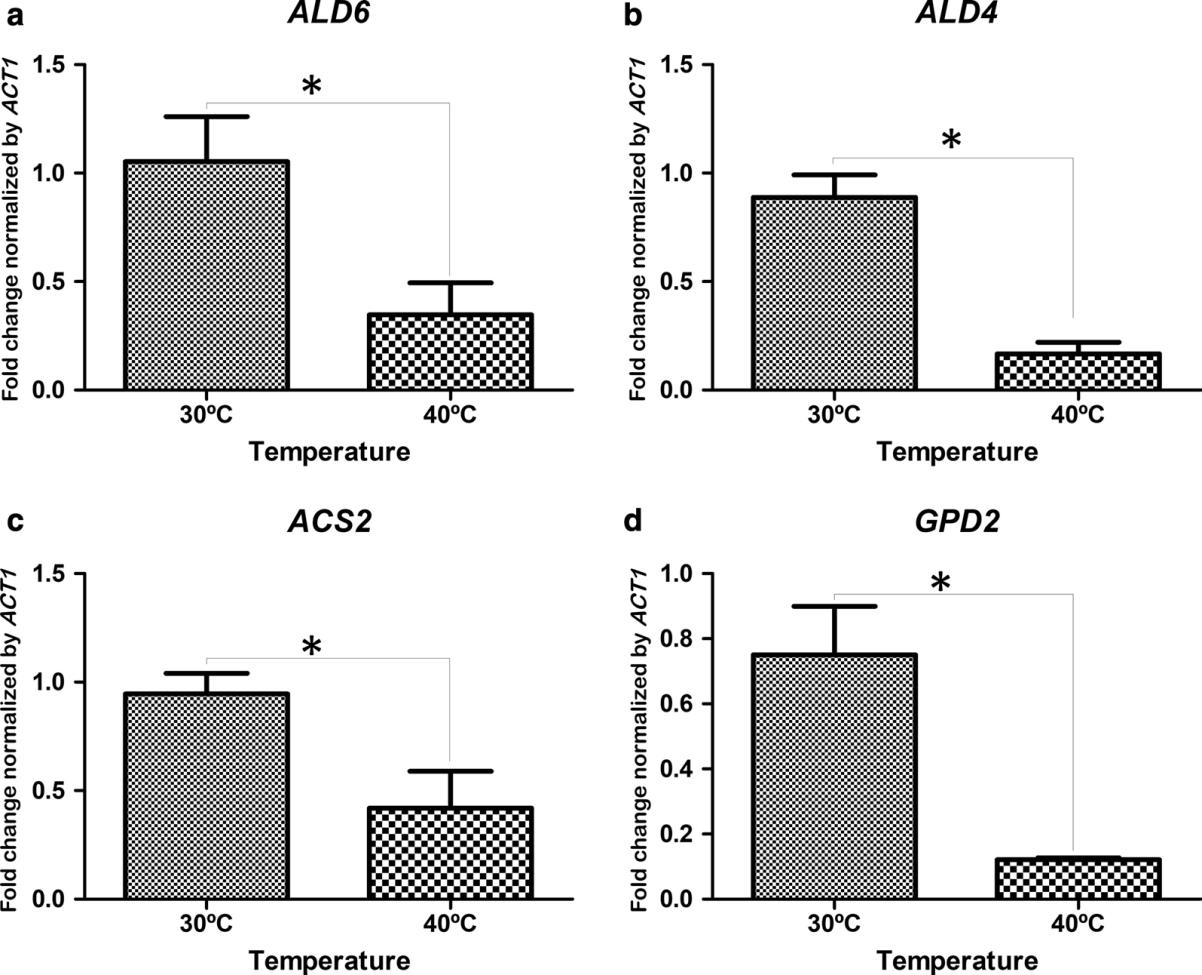
Expression of genes involved in secondary product formation during the fermentative process. mRNA values of aldehyde dehydrogenase-6 (*ALD6*) (**a**), aldehyde dehydrogenase-4 (*ALD4*) (**b**), acetyl CoA synthetase (*ACS2*) (**c**), and glycerol-3-phosphate dehydrogenase 2 (*GPD2*) (**d**) were normalized using beta actin (ACT1) expression. Fermentation assays were performed in duplicate with LBGA-01 at 30 and 40 °C

During fermentation assays, in both glucose-limited chemostats, and in fed-batch fermentation, increase in glycerol production rate and glycerol titer were observed at 40 °C, respectively, when compared to control condition (30 °C). Since GPD1 and GPD2 are key enzymes in glycerol synthesis, we hypothesized that the expression of *GPD1* and *GPD2* would increase during fermentation. Nonetheless, our results indicate that the expression of these genes did not change (Fig. 4). *GPD2* and *GPD1* are paralog genes encoding the isoenzyme NAD-dependent glycerol-3-phosphate dehydrogenase, which has an important role in osmoadaptation (*GPD1*) and anoxic growth conditions (*GPD2*). Mutants lacking both *GPD1* and *GPD2* do not produce detectable glycerol, leading to an accumulation of dihydroxyacetone phosphate (DHAP). This DHAP can be converted to methylglyoxal, a cytotoxic compound that can inhibit yeast growth [17]. Differently, the growth of the LBGA-01 was not affected in either temperature. Interestingly, our results show that the expression of *SNF1* was highly increased in fermentations using the LGBA-01 strain under high temperature (40 °C) (Fig. 4). The kinase *SNF1* was described as an repressor of *GPD2* via phosphorylation to halt glycerol production when nutrients are limited [18]. Therefore, we hypothesize that the unchanged *GPD2* abundance occurs because its repression is exacerbated in the LBGA-01 strain, due to an increase of *SNF-1* expression (Fig. 5d).

As expected, a decrease in *SUC2* (invertase) expression was observed in all strains after 4 h of fermentation (Fig. 4). This was due to the inhibition of *SUC2* expression caused by the accumulation of glucose and fructose during the first hours of fermentation, as a result of the invertase activity [19]. Different patterns were observed for CAT-1 and LBGA-01 after 8 h of fermentation. During CAT1 fermentation, the *SUC2* gene is reactivated as glucose concentration decreases, and the invertase resumes the metabolism of the residual sucrose. The metabolic shift caused by glucose concentration and by inactivation and reactivation of *SUC2* is accompanied by the expression of *SNF1.* The activation of this kinase is glucose-dependent and directly related to the inactivation of glucose transporters, and activation of genes involved in the use of alternative carbon sources [19–21]. As previously described, *SNF1* expression in the LBGA-01 strain is maintained at high levels during the fermentation. Meanwhile, *SUC2* expression decreases, as mentioned above, while sucrose consumption remains unchanged (Fig. 2e, f). In fact, the ethanol production rate is higher at 40 °C than at 30 °C in this strain, accompanying the *SNF1* expression that is increased at fermentation at 40 °C. We suggest that this process happens because there is an augment in internalization of sucrose by MAL31 and AGT1 transporters, since both proteins are able to actively transport sucrose, maltose, and maltotriose, although this process naturally occurs in the absence of glucose [22, 23]. Our hypothesis is also supported by the expression of the *SNF1* gene that is highly expressed in the LBGA-01 strain during the whole fermentation process, even in the presence of glucose, thus possibly activating receptors [24–26]. Interestingly, the expression of SNF1 was higher in the middle and in the end of fermentation conducted with LBGA-01 in both temperatures, when sucrose was used as carbon. Therefore, we argue that LBGA-01 can be used in higher concentrations of this sugar, since sucrose would be inverted by SUC2 and transported by AGT1 at the same time, and later inverted by intracellular SUC2.

When the expression of genes involved in the formation of secondary products of fermentation such as glycerol (*GPD2*), acetate (*ALD6* and *ALD4*), and acetyl-CoA (*ACS2*) was evaluated, we found repression of all of these genes at 40 °C (Fig. 5a–c). This suggests that the alternative pathways for glucose use are inhibited at high temperatures in the LBGA-01 strain, which preferentially uses the available carbon source in ethanol production pathways.

### Quantitative physiological parameters of LBGA-01 during anaerobic glucose-limited chemostat at high temperature

Chemostat cultivations have been broadly applied on quantitative studies of physiological parameters in *S. cerevisiae*. We chose to investigate the impact of high temperature (40 °C) on the anaerobic physiology of LGBA-01 in comparison to control temperature (30 °C). Also, we wished to compare the results with the ones from the experiment conducted by Della-Bianca et al. [27] that used the industrial strain PE-2, largely used for Brazilian ethanol production, using glucose-limited chemostat cultures (Table 1). An advantage of studying microbial cells under continuous culture instead of batch culture is that in the former, the specific growth rate can be held constant under different stressful conditions [28].

**Table 1:** Physiology of *Saccharomyces cerevisiae* strains in glucose-limited conditions

*S. cerevisiae* strain	LBGA-01 (this work)		*Saccharomyces cerevisiae *PE-2 [27]
Temperature	30 °C	40 °C	30 °C
*q* glucose	− 5.28 ± 0.50	− 7.22 ± 0.93	− 5.06 ± 0.15
*q* CO_2_	7.98 ± 0.69	12.02 ± 1.04	8.51 ± 0.28
*q* Ethanol	8.79 ± 1.03	11.50 ± 1.72	7.70 ± 0.26
*q* Glycerol	0.89 ± 0.22	1.38 ± 0.32	0.89 ± 0.04
*q* Lactate	0.05 ± 0.03	0.11 ± 0.02	0.05 ± 0.00
*q*Pyruvate	0.01 ± 0.00	0.08 ± 0.03	Not reported
*q*q Acetate	0.00 ± 0.00	0.01 ± 0.02	0.00 ± 0.00
*X *(g DW L^−1^)	2.64 ± 0.30	2.09 ± 0.26	2.63 ± 0.01
*Y*_X/S _(g DW g glucose^−1^)	0.11 ± 0.01	0.08 ± 0.01	0.11 ± 0.00
*Y*_ETH/S _(g ethanol g glucose^−1^)	0.43 ± 0.12	0.41 ± 0.01	Not reported
*Y*_G/S _(g glycerol g glucose^−1^)	0.09 ± 0.01	0.10 ± 0.01	Not reported
Residual glucose (mM)	0.17 ± 0.23	2.4 ± 0.58	Not reported
*C* recovery (%)	101.97 ± 1.73	101.28 ± 1.35	100.9 ± 0.7

The assays were conducted using anaerobic chemostats with synthetic medium at a dilution rate of 0.1 h^−1^. Specific *q* rates are given in mmol g^−1^ h^−1^. Data are the average value from duplicate or triplicate experiments ± deviation of the mean.

In anaerobic glucose-limited chemostat cultures of the LGBA-01 strains, carbon was mainly diverted to ethanol and CO_2_, and minor amounts of glycerol and lactic acid with a concomitant formation of yeast biomass were produced. When comparing the data obtained from LBGA-01 strain cultivated at 40 °C and 30 °C (control), we observed an increase in consumption of glucose (38%), as well as in the production rates of CO_2_ (51%), glycerol (54%) and ethanol (36%). Differently, we observed a substantial decrease in biomass yield (25%), and no effect on glycerol yield or on maximum specific growth rate during the batch phase (Table 1). Interestingly, we did not observe difference between 40 °C and the control condition in ethanol yield during steady-state in cultures of the strain LGBA-01. In contrast, we observed that glucose concentration was higher at 40 °C during steady state, suggesting a possible inhibition of glucose uptake.

Stressing conditions such as high temperature can generate perturbations in the redox balance inside cells [29]. The increase in rate of synthesis of by-products (such as acetate and lactate) involved in the reoxidation of NADH, are indicative of how cells are responding to this stressful condition, as well as the differential gene expression of the *ACS2* gene reported above.

A similar experimental setup was used by Bianca et al. [31] with the industrial *S. cerevisiae* strain PE-2, known to be highly stress-tolerant [8]. Results obtained in the present study showed that LGBA-01 presents higher ethanol and glycerol production rates than *S. cerevisiae* PE-2 under similar conditions, i.e., at 30 °C. The data suggest an advantage of its use on the industrial process. Moreover, as mentioned by Bianca et al. [31] the absence of acetic acid in all cultivations is a remarkable phenotypic characteristic found in a strain that grows in acidic environments, such as those found in the industrial ethanol process.

A previous report analyzed the thermotolerance of industrial *S. cerevisiae* strains isolated from Brazilian ethanol plants, such as CAT-1, PE-2, BG-1, and JP-1 in synthetic media with glucose as the sole carbon and energy source [14]. Although the authors used different conditions from those reported in this study during the batch phase, i.e., oxygen-limited shake-flask cultures as opposed to anaerobic bioreactors, the growth rates of some strains (JP-1 and CAT-1) were higher at 37 °C than at 30 °C (0.39 and 0.38 h^−1^, respectively). Furthermore, they were lower than the growth rates obtained for the LBGA-01 strain at both 30 °C and 40 °C. With respect to ethanol yield, JP-1 and BG-1 presented an increase in cultivations at 37 °C when compared to 30 °C, differently from our results. Instead, PE-2 presented a small increase in ethanol yield at 37 °C than at 30 °C.

In terms of specific ethanol production rate, our results revealed that LBGA-01 strain has a higher rate at 40 °C than at 30 °C, although reaching similar values of ethanol yield under both conditions. Similarly, increased specific rates of glycerol production were also observed under such conditions, although glycerol yield was not affected. The diversion of carbon away from biomass formation at 40 °C seems to be due to pyruvate and lactate production. This result can be explained by the reduced expression of genes encoding enzymes responsible for the production of secondary products (Fig. 5).

### Fermentative performance of LBGA-01 in conditions mimicking the Brazilian industrial ethanol process under high temperature

Different aspects of *S. cerevisiae* strains such as specific growth, yields in ethanol, glycerol, and cell productivity are commonly investigated under laboratory conditions, using a batch mode operation and synthetic defined culture media offering conditions containing all nutrients in adequate amounts to enable maximum growth rate. In synthetic medium, the carbon source is usually a limiting nutrient [30].

Industrial conditions are not reproducible and vary from batch to batch. There is insufficient data reported for conditions that reproduce the different characteristics found in industrial environments. Specifically in Brazilian 1G ethanol production, sugarcane juice and molasses are often used as a lower cost carbon source for fermentation [31]. Its composition and quality also vary among different batches and harvesting periods; therefore, synthetic laboratory medium replicates of conditions are poor and may lead to misinterpreted conclusions [32]. Also, other stresses are associated with industrial production, such as toxicity of products, non-aseptic conditions, substrate inhibition, cell recycling, acid treatment, bacterial contamination, and temperature stress [30]. Thus, high tolerance for such a great variety of stressful conditions is a desirable feature for a yeast strain in the fuel ethanol industry [30, 33, 34].

To evaluate and study physiological aspects and performance of LBGA-01 under highly stressful conditions, we scaled down the Brazilian 1G ethanol production with sugarcane molasses as carbon source, using a protocol described by Raghavendran et al. [30]. Thermotolerance was investigated by submitting cells to 34 °C and 40 °C, both are unusual laboratory temperatures but commonly found in Brazilian sugarcane mills and inside the reactors due to exothermic reactions of ethanol production [32].

Brazilian 1G ethanol process is operated in fed-batch with yeast cell reuse for the conversion of sugars from molasses into ethanol. The feeding time of molasses lasts between 4 and 6 h, and the fermentation ceases within 6–10 h. The final vat contents are then centrifuged, yielding the “wine” or “beer” and a concentrated yeast cell suspension with 60 to 70% cells (wet weight). The concentrated yeast cell suspension is diluted with water and undergoes an acid treatment at pH 1.8–2.0 by the addition of sulphuric acid to reduce bacterial contamination. After the acidic treatment the cell suspension is recycled to fill another fermentation vat [36].

The protocol reported by Raghavendran et al. [34] encompasses all features of the Brazilian ethanol industry. It allows the evaluation of the main technological results such as fermentation capacity, ethanol and glycerol yields, biomass variation, and viability.

Fermentation capacity was monitored by plotting produced CO_2_ per gram of wet biomass as a function of fermentation time. As shown in other studies, there could be an increase or decrease in biomass, and the plot of total CO_2_ against time could not represent well the fermentation capacity [30]. In this case, normalization of the specific mass is necessary.

**Fig.6:**
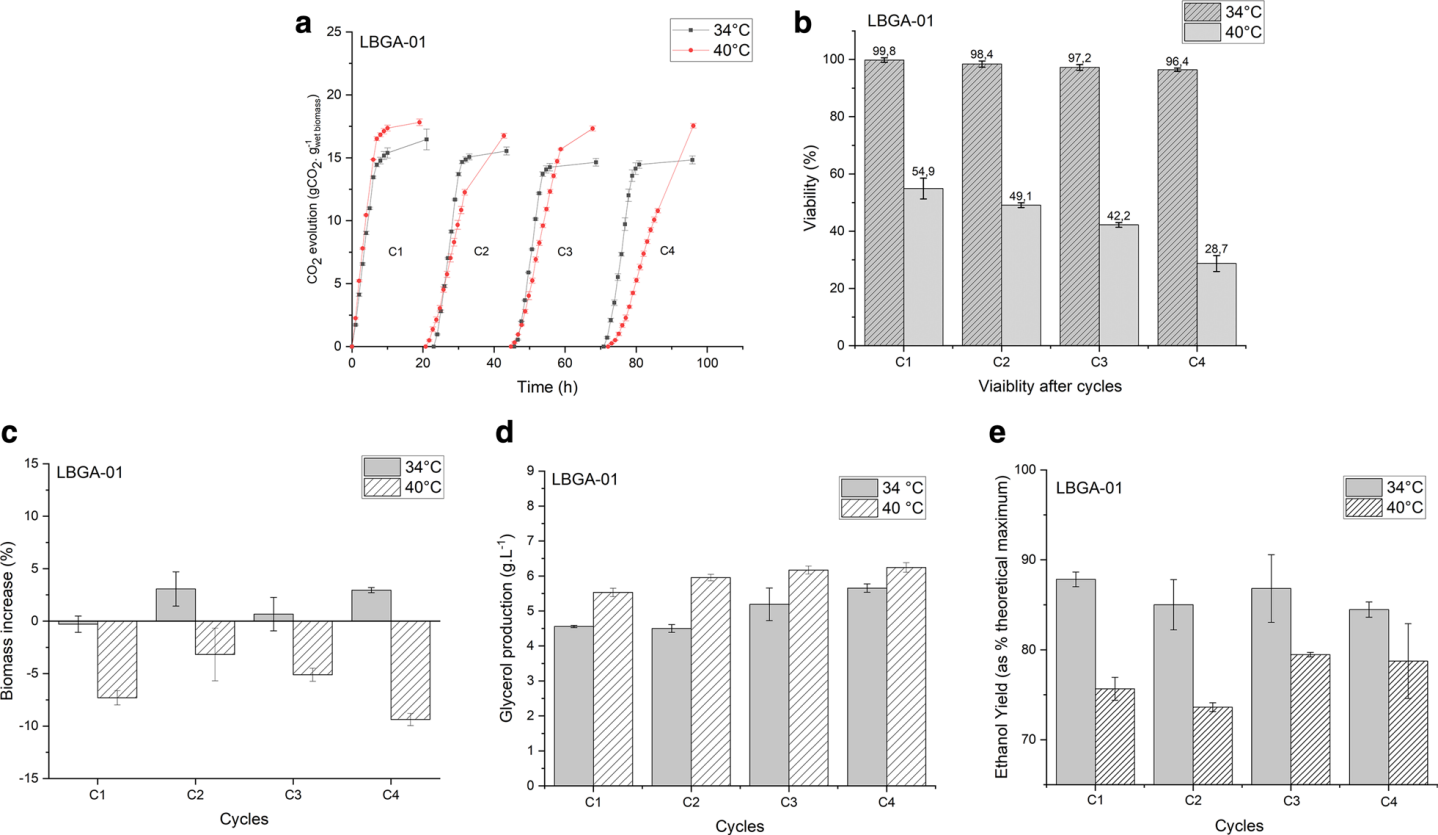
Physiological aspects of LBGA-01 strain in bioreactor fermentation under highly stressful conditions using sugarcane molasses as carbon source. The CO_2_ profiles were normalized by the wet biomass. Cell viability was evaluated over four cycles. Biomass increase was calculated between two consecutive cycles for LBGA-01 at 34 °C and 40 °C. Glycerol production (% w.v^−1^) was evaluated at 34 °C and 40 °C, and ethanol yield is presented as a percentage of theoretical maximum (%)

The fermentation at 34 °C showed a slightly lower fermentation capacity in the first cycle (Fig. 6a) compared to fermentation at 40 °C. At both temperatures, yeast started with virtually the same viability of approximately 80%. The fermentation capacity of the first cycle at 34 °C was kept in the other sequential cycles, hence with viability of about 100% (Fig. 6b). The fermentation capacity decreased from cycle to cycle at 40 °C, as it can be seen from the reduction of the experimental data slope, probably associated with a reduction of viability. At 40 °C, LBGA-01 viability slightly decreased after the first cycle (54.9%), reaching 28.7% of viability after the fourth cycle.

During experiments, both fermentations started with similar yeast biomass (4 ± 0.06 g). Corroborating constant viability, the biomass at 34 °C had a negligible decrease at first, followed by a small increase (less than 5%) in all subsequent cycles (Fig. 6c), resulting in a total biomass increase of 7%. At high temperature, biomass decreased, similarly to viability, with a mean decrease of 6.2% per cycle, resulting in a total reduction of 24% (Fig. 6c).

Although there were differences in biomass variation between cycles, the results at 34 °C corroborate previous results for PE-2 in a scaled-down process [34]. At 40 °C, however, the induced temperature stress resulted in viability loss and, consequently, biomass decrease in each cycle.

Ethanol yield and glycerol production were also assessed during fermentation. As mentioned above, glycerol production is associated with a part of the physiological response of cells to osmotic shock. Thus, its formation occurs in many stressful situations. As a cell response, intracellular glycerol is thought to decrease water activity in the cytosol, leading to higher water uptake [35]. Glycerol levels were monitored and showed an increase every cycle in both temperatures, and were higher at 40 °C than at 34 °C. As a protection mechanism, increased glycerol levels are caused by the high-temperature stressful condition that cells are submitted to (Fig. 6d).

Ethanol yield for each cycle was calculated as described elsewhere [31]. A correction factor for high cell density was applied as previously reported, and a specific volume of 0.7 mL g^−1^ (wet basis) was considered for yeast cells. Thus, the ethanol yield accounts for ethanol from centrifuged *“vinho”* and pelleted yeast biomass. A mass balance for ethanol is applied as the difference between ethanol content at the end of the cycle and ethanol in the beginning (returned *“vinho”* plus pelleted yeast biomass from the previous cycle). Ethanol yield is expressed as a percentage of the maximum theoretical ethanol that could be produced by the total sugar content (Eq. 1):

1 $$ {\text{Ethanol}}\;{\text{yield}}\;(\% )\; = \;\left( {\frac{{10000}}{{51.11\;*\;V_{s} \;*\;TRS}}} \right)*\;\left\{ {\left( {V_{w} \; + \;0.7\;*\;P} \right)\;*\;ET\;\;\left( {V_{v} \; + \;0.7\;*\;P} \right)\;*ET_{P} } \right\} $$

where *V*_*s*_ is the total amount of substrate (mL) of concentration TRS (*g*.100 mL^−1^), 51.11 *g*_ethanol_ (100 g_TRS_)^−1^. *V*_*w*_ is the volume of centrifuged “*vinho*” (mL), *P* is the pelleted yeast biomass (*g*), ET is the ethanol concentration in centrifuged “*vinho*” (% *w* * *v*^−1^). *P*_*p*_ is the pelleted yeast biomass from the previous cycle, ET_*p*_ is the ethanol concentration from centrifuged “*vinho*” from the previous cycle, and *V*_*v*_ is the volume of “*vinho*” from the previous cycle.

Ethanol yields were similar in the four cycles at 34 °C, with a median value of 86.03 ± 1.56%. It is noteworthy that the results obtained for LBGA-01 are similar to those obtained for CEN.PK 113-7D, Baker’s yeast, and S288c, that ranged from 86 to 92% [30]. Moreover, the results are also comparable to those obtained for the two main industrial strains PE-2 (87.2 ± 3.9%) and Ethanol Red™ (87.6 ± 5.1%), both employed in Brazilian 1G production [14, 31]. As previously discussed, fermentation capacity was reduced after cycles at 40 °C, even though ethanol yield after cycles were slightly lower than those obtained at 34 °C, with a mean value of 76.9 ± 2.72% (Fig. 6e). This reduction might be explained by the deviation for the production of other compounds in the fermentative metabolism, such as glycerol production, and also by a higher loss of ethanol attributed to temperature elevation.

Collectively, these results show that LBGA-01 has a good fermentative performance. However, this yeast needs to improve its viability along cell recycles to be used in the Brazilian ethanol production. Efforts using adaptive evolution will be attempted to fix genetic characteristics and increase viability of this yeast in the recycle of high-temperature fermentation.

## Conclusions

We have reported the characterization of a new *S. cerevisiae* strain resident with important fermentative characteristics. In addition, these isolated strains may have important biotechnological characteristics, such as the ability to grow under stress conditions, such as high concentrations of ethanol and sugar, as well as high temperature. Our results showed that although there was a decreasing viability throughout recycling of yeast, the LBGA-01 strain is a potential thermotolerant strain producing a high yield of ethanol at 40 °C. Furthermore, this strain changes its metabolic pathways to resist several stressors produced in 1G and 2G ethanol production, including high ethanol and sugar concentrations, generating better ethanol yield and cell viability for acetic acid, lactic acid, furfural concentrations. These results contribute to the development of production processes using higher temperatures, reducing the use of cooling water during the process, and facilitating the persistence of these strains throughout fermentation, since few wild yeast strains can grow under such conditions. With this manuscript, we hope to encourage new discoveries for the application of LBGA-01 in sugar cane mills.

## Material and methods

### Yeast isolation and identification of thermotolerant yeasts

The yeast strains used in this study were obtained from fermentation tanks after acid treatment, at the São Luiz sugar cane plant located in the city of Ourinhos SP, Brazil. Samples were collected and stored in sterile conical flasks, taken to the laboratory and centrifuged at 3000 rpm for 5 min. The precipitate was washed 3 times with sterile water, and after the last wash, 5 g of the precipitate was eluted to a volume of 50 mL. Serial dilution (1:50; 1: 2500; 1: 12,500) was performed and the yeast was isolated using solid YPD (1% yeast extract, 2% peptone, 2% glucose and 2% agar) incubated for approximately 48 h at 30 °C. After this period, 10 colonies were randomized for analyses.

For cultivation and storage of the strains, a pre-inoculation in 2% YPD (1% yeast extract, 2% peptone and 2% dextrose) liquid medium was performed, followed by growth for 16 h in shaker at 30 ºC, 180 rpm. After growth, 500 µL of culture medium containing *S. cerevisiae* was transferred to flasks containing 500 µL of glycerol 30% (v/v), and kept in a freezer at − 80 °C. All strains were genotyped as described below, and evaluated for growth at high temperatures using the dropout analysis with serial dilutions from 10^6^ to 10^3^ cells mL^−1^.

### DNA extraction

Yeast DNA of all thermotolerant strains found in this study was extracted using phenol–chloroform protocol. In summary, cells were grown overnight in 2% liquid YPD. After this period, cells were centrifuged and lysed with glass beads in 500 µL extraction buffer (200 mM Tris–HCl, 25 mM EDTA and 0.5% SDS), following Malavazi and Goldman [36]. Subsequently, 400 µL of phenol chloroform (50–50%) was added, and sample was centrifuged for 10 min at 13,000 rpm. DNA was precipitated using 600 µL of isopropanol and washed with 300 µL of 70% ethanol. DNA was eluted in 80 µL of water and stored at − 20 °C for subsequent analyses.

### Molecular identification for individual characterization of isolated strains and classification by species

In order to uniquely identify thermotolerant strains, genotyping analysis was performed by PCR using specific primers that were developed based on the amplification of polymorphic regions [37]. To identify yeast species, PCR experiments were performed to amplify the DNA fragment between intergenic regions ITS-1 and ITS4, using specific primers as described by Uranská et al. and other authors [11, 38]. The amplification product (PCR) was sent out for sequencing, and results were compared using BLASTN tool (https://blast.ncbi.nlm.nih.gov/Blast.cgi), as well as matching fragment size results amplified.

### Growth profile of thermotolerant yeasts

Growth tests of thermotolerant and control strains were performed in 50 mL of 2% YPD medium in 250-mL flasks with OD_600nm_ from 0.1 to 30 ºC and 40 ºC, with shaking at 180 rpm for 6 h. Every 2 h, an aliquot was taken and OD_600nm_ measured for the construction of the growth curve. Results were obtained from biological triplicates.

### Fermentation assays using thermotolerant strains

To investigate the fermentative ability of thermotolerant cells, isolated yeasts were submitted to fermentation tests using 4 or 8% glucose as fermentative substrate, at two distinct temperatures, 30 and 40 °C. Yeasts were inoculated in fermentative medium (50 mL) in 250-mL conical flasks with final optical density at 600 nm (OD_600nm_) of 0.1 to ensure standardized inoculations, both temperatures without agitation. Samples were collected at 2-h intervals for glucose consumption analyses. Sugar was measured by glucose oxidase colorimetric enzymatic method (glucose GOD-PAP), following the manufacturer's recommendations.

### Characterization of yeast cell stress resistance (ethanol, sugar, acetic acid, lactic acid, furfural, HMF)

For each stressor, concentrations were established according to the literature. Concentrations higher than those described were used to evaluate the resistance of the thermotolerant yeast LBGA-01 in comparison to the industrial yeast CAT-1 and the laboratory haploid yeast Sc-9721 (MATa *his 3-D200 URA 3–52 leu2D1 lys 2D202 trp 1D63)* (Table 2).

**Table 2:** Stressor concentration used in this study

Stressor	Concentration	Reference
Ethanol	16%	[39]
Sucrose	30%^a^	[40]
Acetic acid	2%	[41]
Lactic acid	4%	[42]
HMF	40 mM	[43]
Furfural	0.9 mM	[43]

^a^In this case, the used medium was only composed of YP2X (2% yeast extract and 4% peptone), adding the desired glucose concentration

Strains were maintained in logarithmic proliferation phase overnight, at 30 °C with agitation of 180 rpm, then diluted to an OD_600nm_ of 0.1 in 50 mL of YPD medium (1% yeast extract, 2% peptone, and 2% dextrose) containing the evaluated stressor at the desired concentration. Assay was performed in triplicate using Erlenmeyer of 250 mL, incubated at 30 °C, 180 rpm for 8 h. Every 2 h, an aliquot of culture medium was removed and the absorbance measured at OD_600_. Experiments were carried out in triplicate.

### qPCR analysis

Total RNA was extracted using Trizol reagent (Invitrogen, Rockville, MS, USA), according to the manufacturers’ protocol. Samples were quantified using a Nano Vue ND-1000 spectrophotometer (GE Healthcare, Chicago, Illinois, USA).

RNA samples (1 μg) were subjected to DNAseI treatment (Invitrogen, Rockville, MS, USA) and reverse transcribed with High Capacity cDNA Reverse Transcription kit using oligo dTV and random primers blend (Thermo Scientific, Waltham, Massachusetts, USA). Primers were designed using the PrimerExpress™ program (Applied Biosystems, Foster City, CA, USA) (see Additional file 3). The concentration of each primer was determined (the best concentration was 150 nM for all primers used in this study) and the amplification efficiency was calculated according to the equation *E* ^(−1/slope)^ to confirm the accuracy and reproducibility of the reactions. Amplification specificity was verified by running a dissociation protocol. qPCRs were performed in a StepOne Plus Real-time PCR System (Thermo Scientific Waltham, Massachusetts, USA). The fold change in mRNA abundance was calculated using 2^−ΔΔCt^ [28] and all values were normalized as the expression of the beta actin (ACT1) gene.

### Chemostat cultivations

Chemostat cultivations with *S. cerevisiae* LBGA-01 strain were carried out in a 2.0-L water jacketed model Labfors 5 (Infors AG, Switzerland), with 1.0 L working volume kept constant by a mechanical drain controlled by a peristaltic pump. Culture medium composition for all cultivations was the one described by Verduyn et al. [44], containing glucose as carbon source, ammonium sulfate as nitrogen source, and supplemented with ergosterol and unsaturated fatty acids in the form of Tween 80, which were dissolved in boiling 96% (v/v) ethanol to final concentrations of 0.01 and 0.42 g L^−1^, respectively [45, 46]. All chemostat cultivations were carried out under anaerobic condition, which was controlled by constant flush of industrial nitrogen gas.

Agitation frequency was set to 800 rpm, temperature was controlled at 30 °C, and pH was controlled at 5.0 via controlled addition of 2 M KOH solution. Precultures for batch bioreactor cultivations were grown overnight in an orbital shaker at 30 °C and 200 rpm in 2500-mL shake flasks containing 30 mL of the defined medium, with 20 g L^−1^ initial glucose. After carbon source exhaustion (that was monitored by a sharp drop in CO_2_ concentration in the off-gas), batch cultivation was switched to continuous mode with a fresh medium containing inhibitor compounds, either isolated or combined, in a feeding of 100 mL h^−1^, which corresponded to a dilution rate of 0.10 h^−1^, assuming a working volume of 1.0 L. Chemostat cultures were performed for at least five residence times prior to sampling.

### Fermentation assays mimicking the Brazilian industrial ethanol process

The bench-scale assays of the industrial fermentation were carried out in triplicate using the protocol described to scale down Brazilian 1G ethanol production [30].

#### Pre-culture and propagation

Pre-culture was prepared with one colony of LBGA-01 inoculated overnight in 100 mL of YPD medium (4% glucose, 1% yeast extract, and 2% peptone) under sterile conditions at 30 °C and 200 rpm. The resultant cell suspension was transferred to flask containing 1 L of the propagation medium (1 L, 10° Brix, sugarcane molasses from São Luiz sugar cane mill) and kept under static conditions for approximately 36 h at room temperature. The flask was carefully agitated from time to time to release trapped CO_2_. This step was performed under non-sterile conditions. After 36 h, the decanted cells were separated from the “vinho” by centrifugation (2000*g*, 4 °C, 15 min).

#### Fermentation

The inoculum for the fermentation step was prepared in triplicates in 50-mL centrifuge tubes with 4 g of cells from the propagation step, 6 mL of water and 2 mL of the *“vinho”* yielding a cell suspension that simulated the efficiency of the industrial centrifuges [31]. The fermentation cycles consisted of an addition of 27.75 mL of fermentation medium (19% TRS sugarcane molasses from São Luiz sugar mill) to tubes in three steps (at 0, 2 and 4 h). Tubes were maintained in an incubator at 34 °C under static conditions and were weighed hourly until 10 h, to monitor mass loss of CO_2_. On the following day, final mass of each tube was measured and tubes were centrifuged (2000*g*, 4 °C, 15 min) to separate the final biomass from the *“vinho”*. The 50-mL centrifuge tubes were weighed, to account for biomass increase from cycle to cycle. Acid treatment was carried out after tubes were weighted, by the addition of 2 mL of *“vinho”* and 6 mL of water to the wet cells. Final pH was adjusted to 2–2.5 with 1 N H_2_SO_4_, and tubes were left at room temperature for one hour before the first addition of fermentation medium to start a new cycle. The simulation was assessed in four cycles, representing 4 days. The same procedure was performed at 40 °C in a static incubator, following the same procedure.

## Supplementary information

**Additional file 1: Fig S1.** Molecular characterization of the thermotolerant strains. Four polymorphic regions of SPA2 (P1), PYR3 (P2), MNN4 (P3) and EPL1 (P4), genes were amplified according described by Carvalho Netto [10]. The ITS amplification were done using primers described by Uranska et al. [11].

**Additional file 2: Figure S2.** ITS sequence of the strains used in the manuscript entitled “Physiological characterization of a new thermotolerant yeast strain isolated during Brazilian ethanol production, and its application in high-temperature fermentation”.

**Additional file 3: Table S1.** Primer sequence used in qPCR analysis. FW-forward primer, RV-reverse primer.

## Data Availability

Not applicable.

## Abbreviations

LBGA: Laboratory of biochemistry and applied genetics
UFSCar: Federal University of São Carlos
USP: University of São Paulo
SSF: Simultaneous Saccharification and Fermentation
SUC2: Sucrose transport protein
AGT1: Alpha-glucoside permease
CAT-1: Industrial yeast strain
GPD2: Glycerol-3-phosphate dehydrogenase 2
ALD6: Aldehyde dehydrogenase-6
ALD4: Aldehyde dehydrogenase-4
ACS2: Acetyl CoA synthetase
HMF: Hydroxymethylfurfural
ITS: Internal transcribed spacer
1G: First generation
2G: Second generation
SNF1: Non-specific serine/threonine protein kinase
MAL31: Maltose permease
ACTB: Beta actin
Eq: Equation
YPD: Yeast extract, peptone and dextrose
DNA: Deoxyribonucleic Acid
EDTA: (Ethane-1, 2-diyldinitrilo) tetraacetic acid
SDS: Sodium lauryl sulfate
PCR: Polymerase chain reaction
OD: Optical density
GOD-PAP: Glucose oxidase-phenol amino phenazone
RNA: Ribonucleic acid
cDNA: QPCR: Real-time polymerase chain reaction
KOH: Potassium hydroxide
CO_2_: Carbon dioxide
TRS: Total reducing sugars
H_2_SO_4_: Sulfuric acid
