# Supplementary material for: Physiological characterization of a new thermotolerant yeast strain isolated during Brazilian ethanol production, and its application in high-temperature fermentation
Source: Biotechnol Biofuels. 2020 Oct 27;13:178. doi: 10.1186/s13068-020-01817-6 (PMC7590731; doi:10.1186/s13068-020-01817-6)
Supplement: Supplementary file 1 — Additional file 1: Fig S1. Molecular characterization of the thermotolerant strains. Four polymorphic regions of SPA2 (P1), PYR3 (P2), MNN4 (P3) and EPL1 (P4), genes were amplified according described by Carvalho Netto [10]. The ITS amplification were done using primers described by Uranska et al. [11]. [file 13068_2020_1817_MOESM1_ESM.docx]

Additional file S1

**
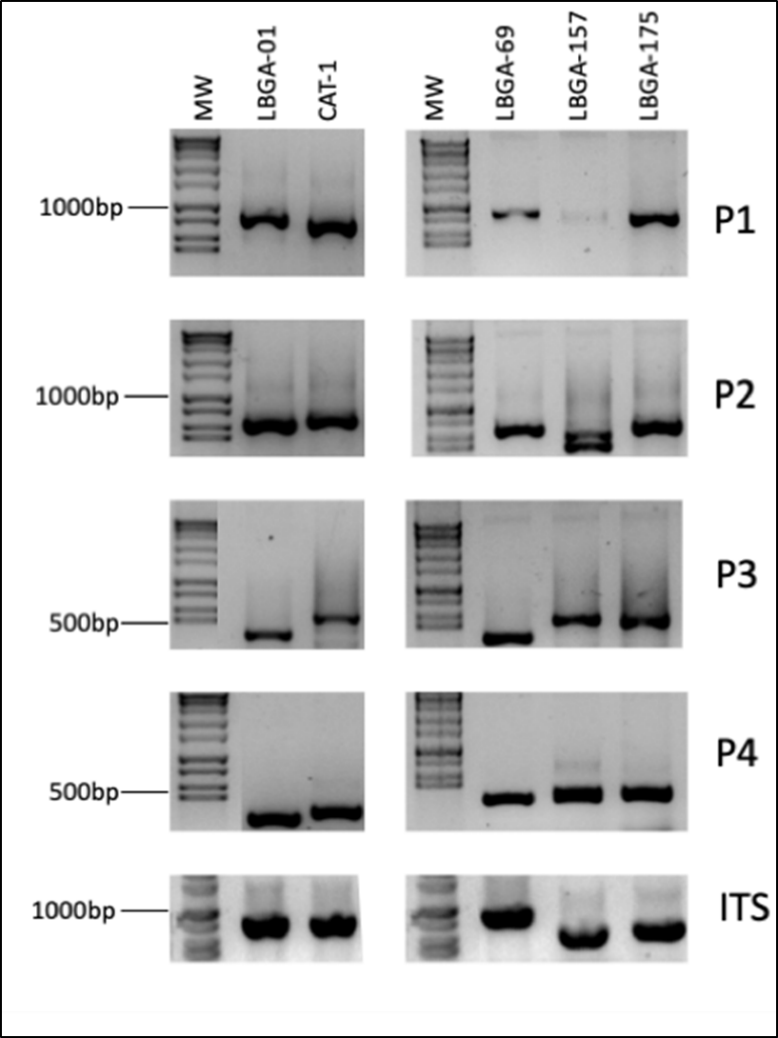
**

Fig S1 – Molecular characterization of the thermotolerant strains. Four polymorphic regions of *SPA2* (P1), *PYR3* (P2), *MNN4* (P3) and *EPL1* (P4), genes were amplified according described by Carvalho Netto [10]. The ITS amplification were done using primers described by Uranska et al. [11].
