# Supplementary material for: Physiological characterization of a new thermotolerant yeast strain isolated during Brazilian ethanol production, and its application in high-temperature fermentation
Source: Biotechnol Biofuels. 2020 Oct 27;13:178. doi: 10.1186/s13068-020-01817-6 (PMC7590731; doi:10.1186/s13068-020-01817-6)

**Figure S3. ITS sequence of the strains used in the manuscript entitled “Global gene expression revealed an increase of HMGB1 and APEX1 proteins and their involvement in oxidative stress, apoptosis and inflammation pathways among beta-thalassemia intermedia and major phenotypes”**

**>LBGA-01**

TTTTGAATGGATTTTTTTGTTTTGGCAAGAGCATGAGAGCTTTTACTGGGCAAGAAGACAAGAGATGGAGAGTCCAGCCGGGCCTGCGCTTAAGTGCGCGGTCTTGCTAGGCTTGTAAGTTTCTTTCTTGCTATTCCAAACGGTGAGAGATTTCTGTGCTTTTGTTATAGGACAATTAAAACCGTTTCAATACAACACACTGTGGAGTTTTCATATCTTTGCAACTTTTTCTTTGGGCATTCGAGCAATCGGGGCCCAGAGGTAACAAACACAAACAATTTTATTTATTCATTAAATTTTTGTCAAAAACAAGAATTTTCGTAACTGGAAATTTTAAAATATTAAAAACTTTCAACAACGGATCTCTTGGTTCTCGCATCGATGAAGAACGCAGCGAAATGCGATACGTAATGTGAATTGCAGAATTCCGTGAATCATCGAATCTTTGAACGCACATTGCGCCCCTTGGTATTCCAGGGGGCATGCCTGTTTGAGCGTCATTTCCTTCTCAAACATTCTGTTTGGTAGTGAGTGATACTCTTTGGAGTTAACTTGAAATTGCTGGCCTTTTCATTGGATGTTTTTTTTTCCAAAAAAAGGTTTCCCTGCCTGCTTGAGGGAAAAAGCAAATACGG

**Blast result:**


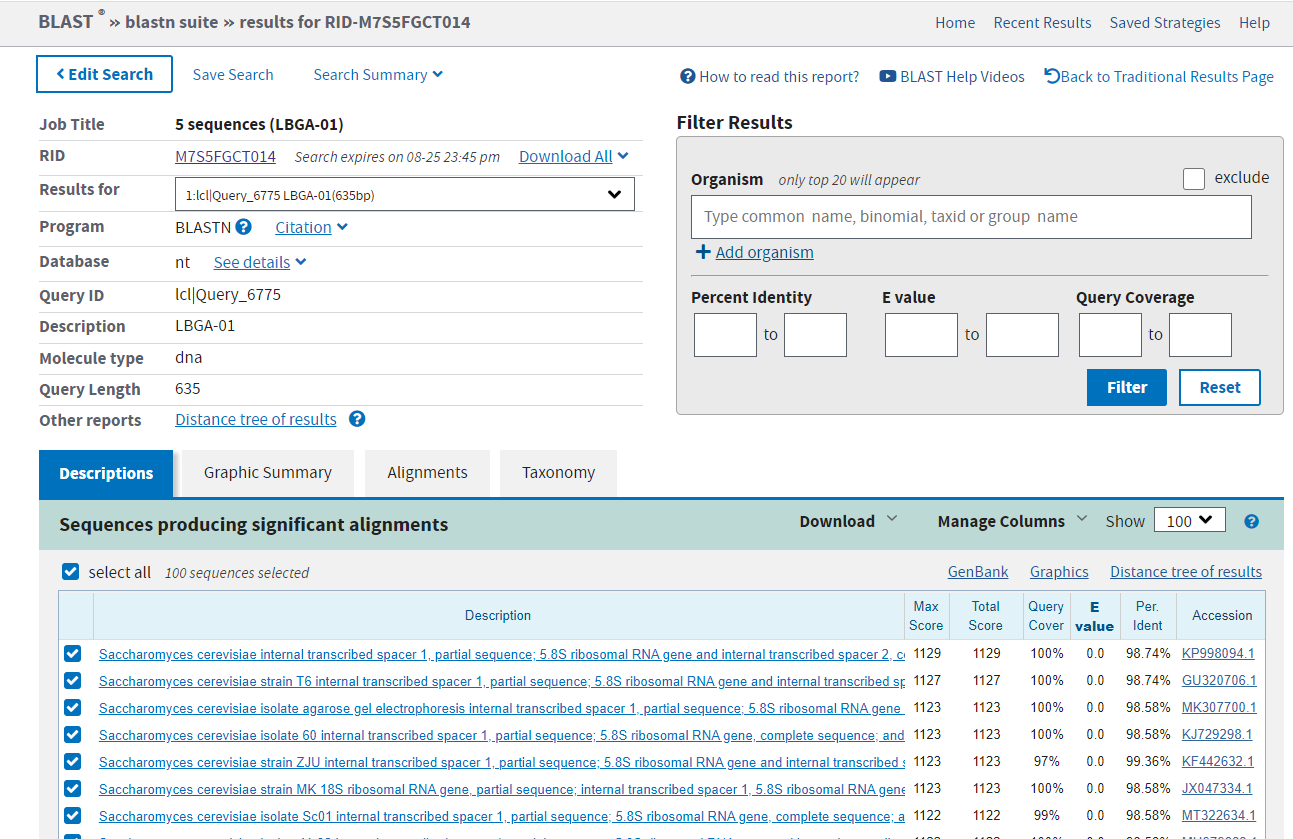


**>CAT-1**

AAGAGCATGAGAGCTTTTACTGGGCAAGAAGACAAGAGATGGAGAGTCCAGCCGGGCCTGCGCTTAAGTGCGCGGTCGTGCTAGGCTTGTAAGTTTCTTTCTTGCTATTCCAAACGGTGAGAGATTTCTGTGCTTTTGTTATAGGACAATTAAAACCGTTTCAATACAACACACTGTGGAGTTTTCATATCTTTGCAACTTTTTCTTTGGGCATTCGAGCAATCGGGGCCCAGAGGTAACAAACACAAACAATTTTATCTATTCATTAAATTTTTGTCAAAAACAAGAATTTTCGTAACTGGAAATTTTAAAATATTAAAAACTTTCAACAACGGATCTCTTGGTTCTCGCATCGATGAAGAACGCAGCGAAATGCGATACGTAATGTGAATTGCAGAATTCCGTGAATCATCGAATCTTTGAACGCACATTGCGCCCCTTGGTATTCCAGGGGGCATGCCTGTTTGAGCGTCATTTCCTTCTCAAACATTCTGTTTGGTAGTGAGTGATACTCTTTGGAGTTAACTTGAAATTGCTGGCCTTTTCATTGGATGTTTTTTTTCCAAAGAGAGGTTTCTCTGCGTGCTTGAGGTATAATGCAAGTACGGTCGTTTTAGGTTTTACCAACTGCGGCTAATCTTTTTTTATACTGAGCGTATTGGAACGTTATCGATAAGAAGAGAGCGTCTAGGCGAACAATGTTCTTAAAGTTTGACCTCAAATCAGGTAGGAGTACCCGCTGAACTTAAGCATATAAAACCCGGAGAGAAAAAGAAGAAAAAAAAAAAATTAAAAAATTTGGAAAAAGGAAATTTTTGTTTGGGGAAGAGCAAGGAGAGATTTTACTGGGGGAAGAAGACAAGAGATGGAGAAGTCCAGCCGGGCCTGCGCTTAAGTGCGCGGTCGTGCT

**Blast result:**


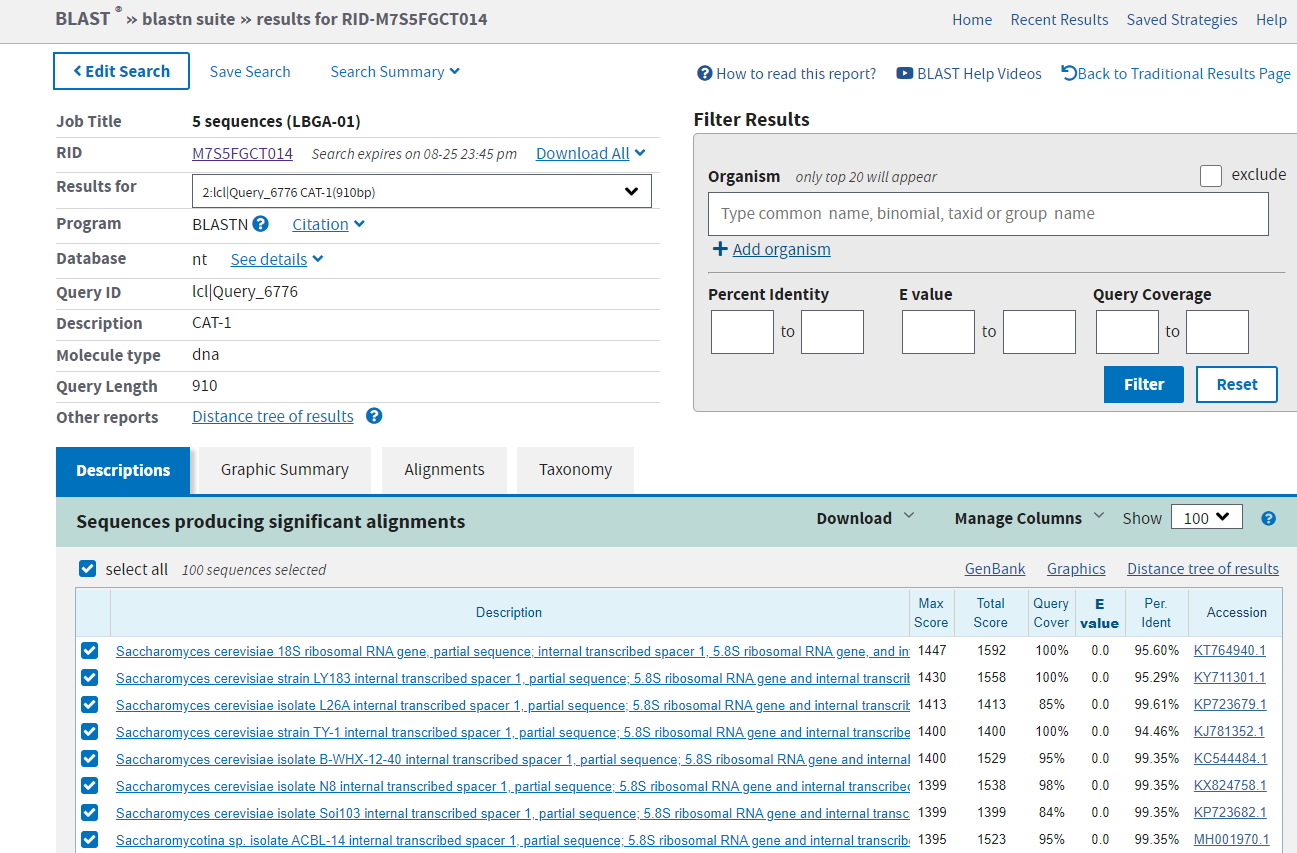


**>LBGA-69**

GGCAAGAGCATGAGAGCTTTTACTGGGCAAGAAGACAAGAGATGGAGAGTCCAGCCGGGCCTGCGCTTAAGTGCGCGGTCTTGCTAGGCTTGTAAGTTTCTTTCTTGCTATTCCAAACGGTGAGAGATTTCTGTGCTTTTGTTATAGGACAATTAAAACCGTTTCAATACAACACACTGTGGAGTTTTCATATCTTTGCAACTTTTTCTTTGGGCATTCGAGCAATCGGGGCCCAGAGGTAACAAACACAAACAATTTTATTTATTCATTAAATTTTTGTCAAAAACAAGAATTTTCGTAACTGGAAATTTTAAAATATTAAAAACTTTCAACAACGGATCTCTTGGTTCTCGCATCGATGAAGAACGCAGCGAAATGCGATACGTAATGTGAATTGCAGAATTCCGTGAATCATCGAATCTTTGAACGCACATTGCGCCCCTTGGTATTCCAGGGGGCATGCCTGTTTGAGCGTCATTTCCTTCTCAAACATTCTGTTTGGTAGTGAGTGATACTCTTTGGAGTTAACTTGAAATTGCTGGCCTTTTCATTGGATGTTTTTTTTCCAAAGAGAGGTTTCTCTGCGTGCTTGAGGTATAATGCAAGTACGGTCGTTTTAGGTTTTACCAACTGCGGCTAATCTTTTTTTATACTGAGCGTATTGGAACGTTATCGATAAGAAGAGAGCGTCTAGGCGAACAATGTTCTTAAAGTTTGACCTCAAATCAGGTAGGAGTACCCGCTGAACTTAAGCATATCATAAACCGGAAGAAAAAGAACAATTAAAAAAATTTAAAAATTATAAAAAGGATTTTTTTGTTTTGGGCAAAATATGAGAGCTTTACGGGCCAAAAAAAAAAAAGAAAGTCACCGGCCCGCCCTTAATGGCCGGTCCAACAAGCCTTGAAAATTTCCTTCTTGGTTTTCCAAAAGGGGAAAAAATTTTTATGCTTTTGTGTATAGGAAAATTAAAAAATTAACCACCATGGGGGATTTCCGTTCTGCACCTTTTCTGGGGTTCAAATAGGGGGCCAAAGGAAAAAACAAAAATTTTTTTTCCTAGAATTTTTC

**Blast result:**


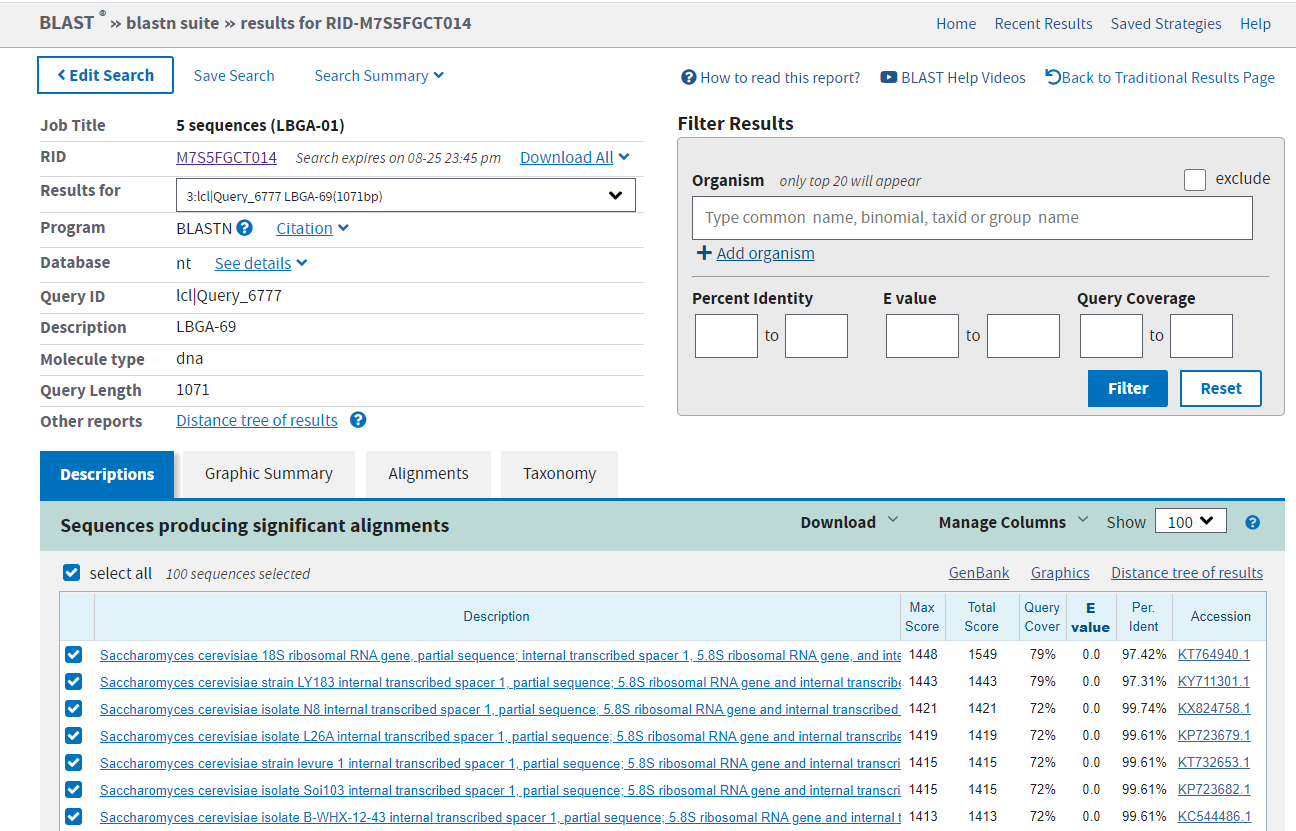


**>LBGA-157**

AAGTTTAGTTAGGCATTTATACAGTGAACTGCGAATGGCTCATTAAATCAGTTATCGTTTATTTGATAGTTCCTTTACTACATGGATATCTGTGGTAATTCTAGAGCTAATACATGCTTAAAATCTCGACCCTTTGGAAGAGATGTATTTATTAGATAAAAAATCAATGTCTTCGGACTCCTTGATGATTCATAATAACTTTTCGAATCGCATGGCCTTGTGCTGGCGATGGTTCATTCAAATTTCTGCCCTATCAACTTTCGATGGTAGGATAGTGGCCTACCATGGTTTCAACGGGTAACGGGGAATAAGGGTTCGATTCCGGAGAGGGAGCCTGAGAAACGGCTACCACATCCAAGGAAGGCAGCAGGCGCGCAAATTACCCAATCCTAATTCAGGCCGCAGAAGGCGGGGAAGGGAATTCCAAGGAGGAGGGGGGGGGGGGGAGGAGAGGGGGGGGGGGGGGGAGGAGGTGGGGAAGAT

**Blast result:**


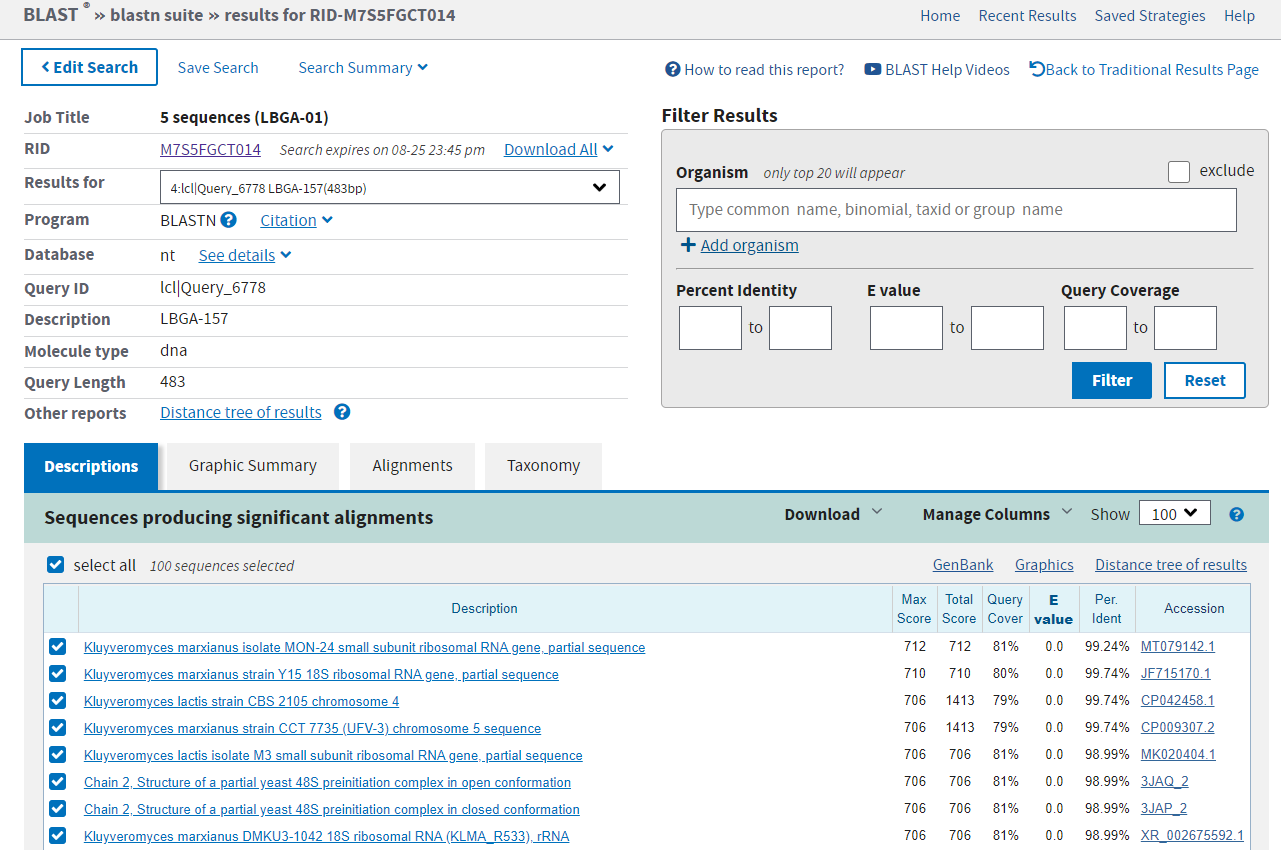


**>LBGA-175**

AAGTTTTAGTATCGCATTTATACAGTGAACTGCGAATGGCTCCATTTAAATCAGTTATCGTTTATTTGATAGTACCTTTACTACTTGGTATAACCGTGGTAATTCTAGAGCTAATACATGCTAAAAACCCCGACTGTTTGGAAGGGGTGTATTTATTAGATAAAAAATCAATGCTCTTCTGAGCTCTTTGATGATTCATAATAACTTTTCGAATCGCATGACTTCGTGTCGGCGATGGTTCATTCAAATTTCTGCCCTATCAACTTTCGATGGTAGGATAGTGGCCTACCATGGTTTCAACGGGTAACGGGGAATAAGGGTTCGATTCCGGAGAGGGAGCCTG

**Blast result:**


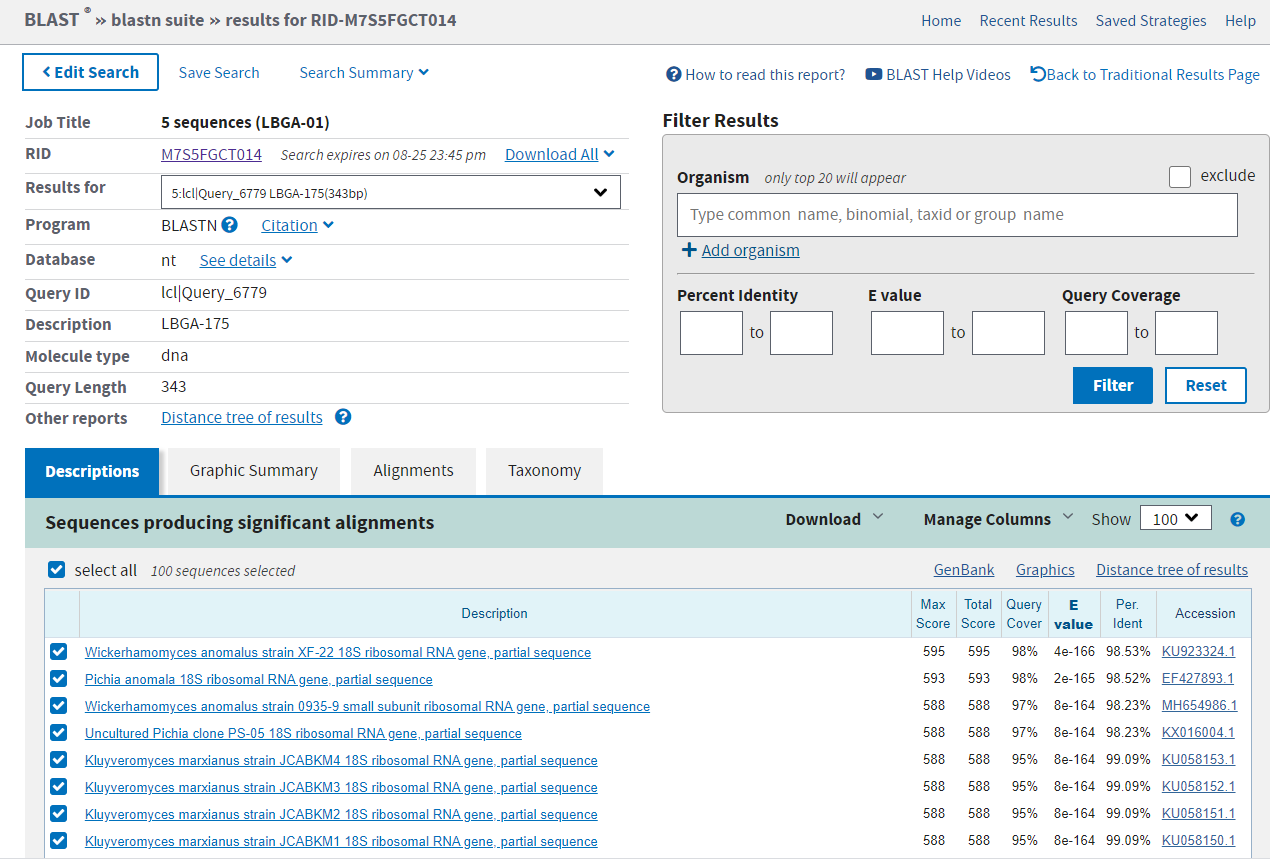

Supplement: Supplementary file 2 — Additional file 2: Figure S2. ITS sequence of the strains used in the manuscript entitled “Physiological characterization of a new thermotolerant yeast strain isolated during Brazilian ethanol production, and its application in high-temperature fermentation”. [file 13068_2020_1817_MOESM2_ESM.docx]
