# Supplementary material for: Physiological characterization of a new thermotolerant yeast strain isolated during Brazilian ethanol production, and its application in high-temperature fermentation
Source: Biotechnol Biofuels. 2020 Oct 27;13:178. doi: 10.1186/s13068-020-01817-6 (PMC7590731; doi:10.1186/s13068-020-01817-6)
Supplement: Supplementary file 3 — Additional file 3: Table S1. Primer sequence used in qPCR analysis. FW-forward primer, RV-reverse primer. [file 13068_2020_1817_MOESM3_ESM.docx]

**Table S1**

**Table S1- Primer sequence used in qPCR analysis. FW- forward primer, RV-reverse primer**

| **Gene** | **Primer sequence** | **FW/RV concentration** | ***Treshold*** | **Efficiency %** |
| --- | --- | --- | --- | --- |
| **GPD1** | FW: CATTGCCACCGAAGTCGCTC | 150/150 | 0,1 | 98 |
|  | RV: GCCCTCGCCTCTGAAATCCT |  |  |  |
| **GPD2** | FW: TTCGAGTTGGGCTCCAAGGG | 150/150 | 0,1 | 100 |
|  | RV: ACCAATGCTCCTTGGCCACT |  |  |  |
| **ALD6** | FW: CCTTAGCCCGTGGGGATGTT | 300/300 | 0,097 | 95 |
|  | RV: GCCGTCACCGGTGTTGATTG |  |  |  |
| **ALD4** | FW: GCGGACGCCGAGTTGAAAAA | 150/150 | 0,109 | 98 |
|  | RV: TGAACCCGCACAACAGACCT |  |  |  |
| **ACS2** | FW: TGGTTCTGCTACCGTGCCAT | 150/150 | 0,099 | 96 |
|  | RV: ACGGTCGTGGTGGTTCCAAA |  |  |  |
| **AGT1** | FW: AGGTATGGCCACCGACAAGG | 150/150 | 0,078 | 99 |
|  | RV: GCGCTGCTTCCAGAACCAAA |  |  |  |
| **MAL31** | FW: TGGGACAGGCATTGTGTGGT | 150/150 | 0,2 | 103 |
|  | RV: GTTGACCGAACGCCCAACAT |  |  |  |
| **SUC2** | FW: GGGGCCATGCTACTTCCGAT | 150/150 | 0,1 | 95 |
|  | RV: TCGTTACGCTTGGGAGCGAT |  |  |  |
| **SNF1** | FW: CACAGCACCTGCCAATGCAA | 150/150 | 0,08 | 99 |
|  | RV: CCCCTCTCCCAGCGTTTTGA |  |  |  |
| **HTX1** | FW: GCTGGCAGAATCGACGAAGC | 150/150 | 0,7 | 100 |
|  | RV: GCAGTACCAGCGGCTCTCAT |  |  |  |
| **ADR1** | FW: ACGAGAGCGTTCGCAAGACA | 150/150 | 0,072 | 100 |
|  | RV: GTTGCAGAGGCCACAGGGAT |  |  |  |
| **BACT** | FW: AGGTATTGCCGAAAGAATGC | 100/300 | 0,1 | 99 |
|  | RV: AAGGTAGTCAAAGAAGCAAG |  |  |  |
